# Role of hypertension in the cardiovascular-kidney-metabolic syndrome among black adults: The Jackson Heart Study

**DOI:** 10.1038/s41371-025-01078-6

**Published:** 2025-10-04

**Authors:** Lama Ghazi, Medha Dubal, Alain Bertoni, April Carson, Bessie A. Young, Cora E. Lewis, Chibuike J. Alanaeme, Dayna A. Johnson, Daichi Shimbo, Kathryn Foti, Lisandro D. Colantonio, Milla Arabadjian, Rikki Tanner, Paul Muntner

**Affiliations:** 1 Department of Epidemiology, School of Public Health, University of Alabama at Birmingham, Birmingham, AL USA; 2 Wake Forest School of Medicine, Department of Epidemiology and Prevention, Winston-Salem, NC USA; 3 Department of Medicine, University of Mississippi Medical Center, Jackson, MS USA; 4 Division of Nephrology, Department of Medicine, and Office of Healthcare Equity, University of Washington, Seattle, WA USA; 5 Department of Epidemiology, Emory University Rollins School of Public Health, Atlanta, Georgia; 6 The Columbia Hypertension Center and Lab, Columbia University Irving Medical Center, New York, New York UK; 7 Center for Population and Health Services Research, Department of Foundations of Medicine, New York University Grossman Long Island School of Medicine, Mineola, NY USA; 8 Perisphere Real World Evidence, Austin, TX USA

**Keywords:** Diseases, Cardiovascular diseases, Hypertension

## Abstract

The cardiovascular-kidney-metabolic (CKM) syndrome consists of four progressive stages and is characterized by the interaction of metabolic risk factors, chronic kidney disease (CKD), and cardiovascular disease (CVD). We assessed the prevalence of hypertension in CKM and its role in progression to more advanced stages. We included 2118 Black adults from the Jackson Heart Study without a history of coronary heart disease, heart failure, stroke or stage 0 CKM (normal weight, no metabolic risk factors or CVD) at baseline. Participants were categorized into CKD stage: Stage 1: overweight/obesity, abdominal obesity or dysfunctional adipose tissue without metabolic risk factors or subclinical CVD; Stage 2: metabolic risk factors (hypertension, diabetes, hypertriglyceridemia, metabolic syndrome or CKD); or Stage 3: subclinical CVD. We used Cox proportional hazards regression to estimate the hazard ratio (HR) of developing stage 4 CKM, defined by a CVD event, in participants with hypertension and stages 2 and 3 CKM. At baseline, 20.2, 69.1 and 10.6% of participants had stage 1, 2 and 3 CKM, respectively. Hypertension was the most common metabolic risk factor in participants with stage 2 and 3 CKM with a prevalence of 80 and 95%, respectively. Incidence rates (95%CI) of stage 4 CKM per 1000 person-years were 1.4 (0.4, 2.4) for stage 1 CKM, 7.5 (6.1, 8.9) for stage 2 CKM with hypertension, and 26.6 (19.8, 33.3) for stage 3 CKM with hypertension. The HRs (95% CI) for developing stage 4 CKM were 3.25 (1.56, 6.80) and 5.11 (2.05,12.78) among participants with hypertension and stage 2 and 3 CKM versus stage 1 CKM, respectively. Hypertension was associated with an increased risk for progression to stage 4 CKM among Black adults.

## Introduction

In 2023, the American Heart Association (AHA) published a presidential advisory on Cardiovascular-Kidney-Metabolic (CKM) health that provided a comprehensive approach for the prevention, early detection, and management of CKM syndrome [1]. The CKM syndrome is characterized by the interaction of metabolic risk factors, chronic kidney disease (CKD) and cardiovascular disease (CVD) which lead to multi-organ dysfunction and adverse cardiovascular outcomes. The advisory defined 4 mutually exclusive CKM stages: Stage 1: having overweight/obese, abdominal obesity or dysfunctional adipose tissue, having no other metabolic risk factors and no subclinical or clinical CVD; Stage 2: the presence of metabolic risk factors including hypertension, diabetes, high serum triglycerides, moderate to high-risk CKD, and the metabolic syndrome, without sub-clinical or clinical CVD; Stage 3: the presence of subclinical CVD or high-risk CKD but not clinical CVD among adults with overweight/obese or abdominal obesity or dysfunctional adipose tissue or having one or more metabolic risk factor, and Stage 4: the presence of clinical CVD among adults with overweight/obesity, abdominal obesity, or dysfunctional adipose tissue or having one or more metabolic risk factor [1]. A cross-sectional analysis of the National Health and Nutrition Examination Survey (NHANES) reported almost 90% of US adults met the criteria for stage 1 through 4 CKM [2]. However, that analysis was unable to investigate factors contributing to the transition from stages 2 and 3 CKM to stage 4 CKM. Identifying such factors may allow for directed interventions to potentially prevent clinical CVD.

The prevalence of hypertension is high among US adults [3], and is more common in those with obesity, abdominal obesity and dysfunctional adipose tissue [4, 5]. Compared to their counterparts without hypertension, adults with hypertension have a higher prevalence of subclinical CVD, and increased risk for CKD, clinical CVD, and mortality [4]. Determining the association of hypertension with the progression of CKM syndrome may inform prevention strategies aimed at controlling blood pressure (BP) in earlier stages of CKM. The goal of the current study was to determine the prevalence of hypertension in Black adults with stage 2 and 3 CKM and to assess its association with the progression to stage 4 CKM. For comparison, we determined the prevalence of the other metabolic risk factors in the CKM syndrome including diabetes, high serum triglycerides, moderate to high-risk CKD and the metabolic syndrome, and their association with progression to stage 4 CKM. To accomplish these goals, we analyzed data from the Jackson Heart Study (JHS), a cohort of Black US adults.

## Methods

### Study population

The JHS enrolled 5306 Black men and women from the Jackson, Mississippi metropolitan area to study CVD among Black [6]. After a baseline visit in 2000–2004, follow-up visits were conducted in 2005–2008 and 2009–2013. For the current analysis, we excluded participants who had a history of CHD or stroke at baseline or a history of heart failure before 1/1/2005, when follow-up for heart failure began, and participants who were missing data for covariates at baseline, including age, sex, education, smoking, and alcohol consumption. Additionally, we excluded participants who were missing data on the variables used to define CKM stages, those who did not have data on clinical CVD events during follow up and participants who did not have left ventricular mass index (LVMI) measured at baseline. We excluded participants with CKM stage 0 at baseline (Table S1) or participants who had subclinical CVD at baseline but did not have obesity or overweight, abdominal obesity, dysfunctional adipose tissue or metabolic risk factors as they do not meet the criteria for any CKM stage [1].

#### Ethics approval and consent to participate

Institutional Review Board approval was obtained from the University of Mississippi Medical Center, Jackson State University, and Tougaloo College the University of Alabama at Birmingham, and all participants provided written informed consent. All methods were performed in accordance with the relevant guidelines and regulations.

### Data collection

At baseline, participants completed an in-home interview and study visit where trained staff collected self-reported sociodemographic characteristics, lifestyle behaviors, medication use and medical history [6]. Pill bottles for prescription and over the counter medications taken in the two weeks before the study examination were reviewed and names of medications were recorded and categorized by class.

During the study visit, research staff measured height, weight, waist circumference, BP, and collected blood and urine samples. A description of BP measurement techniques used in the JHS have been previously published [7, 8]. In brief, BP was measured by trained technicians following a standardized protocol [6, 7, 9]. Participants’ right arms were fitted with an appropriately-size cuff. Participants sat upright and rested for at least 5 min with their back and arms supported, feet flat on floor, legs uncrossed. Two BP measurements were performed with 1 min rest in between using a random-zero sphygmomanometer (Hawksley and Sons, Ltd). The JHS Coordinating Center implemented quality control procedures, including monitoring for digit preference by individual staff and evaluating consistency in mean BP readings within and across technicians. Measurements were calibrated to a semi-automated device following a calibration study [8]. These two measurements were averaged for analysis.

Albumin and creatinine were quantified from a 24-hour urine collection or from a spot urine sample using the nephelometric immunoassay and enzymatic methods [7]. Albuminuria was defined as urine albumin to urine creatinine ratio (ACR) > 30 mg/g [10]. Serum creatinine was measured using enzymatic methods and calibrated to isotope dilution mass spectroscopy traceable creatinine [11]. Estimated glomerular filtration rate (eGFR) was calculated using serum creatinine values and the 2021 Chronic Kidney Disease Epidemiology Collaboration (CKD-EPI) non race-specific equations [10]. Echocardiography was performed at the baseline visit by a trained sonographer using standardized protocols (Sonos-4500; Philips Medical Systems) [12, 13]. Left ventricular mass was indexed to body surface area (LVMI). Left ventricular hypertrophy (LVH) was defined as LVMI ≥ 96 g/m^2^ in women and ≥116 g/m^2^ in men [14].

### Definition of CKM stages 1, 2 and 3

CKM stages and dysfunctional adipose tissue were defined according to the 2023 AHA Presidential Advisory as described in the introduction and Table S1 [1]. Hypertension was defined as self-reported antihypertensive medication use, systolic BP (SBP) ≥ 130 mm Hg, or diastolic BP (DBP) ≥ 80 mm Hg.

### Definition of incident stage 4 CKM

Incident stage 4 CKM was defined as having a CVD event including coronary heart disease (CHD), heart failure, or stroke during follow-up. Potential events were identified during annual follow-up interviews and through surveillance of local hospital discharge records. Medical records were adjudicated to confirm diagnoses. Incident CHD was defined as the first occurrence of a definite or probable myocardial infarction, definite fatal myocardial infarction, or definite fatal CHD [15]. A computer-generated diagnosis and physician adjudication were used to classify hospitalized and fatal stroke events and type of stroke event. CHD and stroke events were adjudicated from baseline through December 31, 2016, the last date currently available in the JHS. Incident heart failure hospitalization was identified through annual telephone interviews and hospital discharge lists [15–17]. Heart failure events were adjudicated from January 1, 2005 through December 31, 2016 and events were confirmed through review of medical records by trained medical personnel [15].

### Statistical analysis

We described the demographic and clinical characteristics of participants at baseline with CKM stages 1, 2 and 3, separately, using means with standard deviations and proportions. We calculated the prevalence of overweight/obesity, abdominal obesity, dysfunctional adipose tissue, hypertension, diabetes, hypertriglyceridemia, metabolic syndrome, moderate to high-risk CKD for participants with stage 2 and stage 3 CKM, separately. Next, we calculated the proportion of participants with 1, 2, 3, 4, and 5 metabolic risk factors and combinations of these risk factors among participants with stage 2 and stage 3 CKM, separately.

We estimated the incidence rate of stage 4 CKM for participants with stage 1, 2 and 3 CKM, overall, and with each metabolic risk factor among participants with stage 2 CKM and stage 3 CKM at baseline. Follow-up continued from baseline until incident stage 4 CKM or administrative censoring at the last JHS contact or December 31, 2016, whichever occurred first. We used Cox proportional hazards models to estimate the hazard ratio (HR) for incident stage 4 CKM associated with each metabolic risk factor among participants with stage 2 CKM versus participants with stage 1 CKM. Stage 1 CKM participants, by definition, had no metabolic risk factors. Models were adjusted sequentially: Model 1 included adjustment for age, sex, and education; Model 2 included the variables in Model 1 and smoking and alcohol use; and Model 3 included the variables in Model 2 and BMI. Variables included for adjustment were chosen to account for potential confounders while maintaining parsimony in our modeling approach.

The majority of participants with stage 2 or stage 3 CKM had more than 1 metabolic risk factor, with hypertension being the most common. Therefore, in a *post-hoc* analysis, we calculated the incidence rate of stage 4 CKM for participants with stage 2 and stage 3 CKM, separately, who were (1) without hypertension but with one or more of the other metabolic risk factors, (2) with hypertension and one or more of the other metabolic risk factors and (3) with hypertension but with no other metabolic risk factors. Among these groups, we used Cox models to estimate the HR for incident stage 4 CKM associated with having hypertension with other metabolic risk factors or with having hypertension with no other metabolic risk factors compared to participants without hypertension but who had other metabolic risk factors. We used three levels of adjustment as described above. In secondary analysis, we used Cox models to estimate the HR for developing stage 4 CKM comparing participants with versus without each metabolic risk factor for participants with stage 2 CKM and stage 3 CKM, separately.

## Results

### Characteristics of JHS adults with stage 1, 2 and 3 CKM

Of the 5306 JHS participants, 2118 were included after excluding those with baseline CVD (n = 1025), missing covariates (n = 709), missing LVMI (n = 1187), CKM stage 0 (n = 262), and participants who did not fit the criteria for stage 1, 2 or 3 CKM (n = 5) (Figure S1). At baseline, 20.2, 69.1 and 10.6% had stage 1, 2, and 3 CKM at baseline, respectively (Table 1). Participants with higher stages of the CKM syndrome were older, more likely to have less than high school education, smoke, not drink alcohol, and had higher SBP, DBP, BMI, waist circumference, hemoglobin A1c (HbA1c), fasting blood glucose, serum triglycerides, total cholesterol and had lower eGFR. SBP and DBP distribution by CKM stage are shown in Figure S2 and S3, respectively.

**Table 1:** Baseline characteristics of Jackson Heart Study participants with stage 1, 2 and 3 Cardiovascular-Kidney-Metabolic (CKM) syndrome, N = 2118.

	Cardiovascular-Kidney-Metabolic syndrome stage		
	Stage 1 N = 429 (20.2%)	Stage 2 N = 1464 (69.1%)	Stage 3 N = 225 (10.6%)
**Demographics**			
Age, years	43.8 ± 10.8	52.3 ± 10.6	64.5 ± 11.2
Male sex, n (%)	127 (29.6%)	539 (36.8%)	73 (32.4%)
**Socioeconomic characteristics**			
Less than high school education, n (%)	24 (5.5%)	161 (11.0%)	74 (28.5%)
Current smoking, n (%)	37 (8.6%)	158 (10.7%)	31 (13.8%)
Current alcohol use, n (%)	239 (55.7%)	708 (48.3%)	65 (28.8%)
**Vital signs and anthropometric measurements**			
Systolic blood pressure, mmHg	112.8 ± 8.3	126.9 ± 14.1	139.6 ± 19.7
Diastolic blood pressure, mmHg	71.4 ± 5.6	77.3 ± 8.2	76.0 ± 9.6
Body mass index, kg/m^2^	31.2 ± 6.1	32.3 ± 6.3	34.3 ± 7.5
Waist circumference, cm	96.4 ± 14.1	101.4 ± 14.1	106.9 ± 15.4
**Laboratory values**			
Hemoglobin A1C, n (%)	5.3 ± 0.4	5.8 ± 1.1	6.4 ± 1.3
Fasting blood glucose, mg/dL	86.3 ± 6.7	98.6 ± 28.9	114.4 ± 46.0
Triglyceride, mg/dL	71.6 ± 23.6	112.1 ± 68.2	120.5 ± 95.0
Total cholesterol, mg/dL	187.2 ± 36.2	201.6 ± 37.6	203.1 ± 41.2
High Density Lipoprotein cholesterol, mg/dL	51.6 ± 14.3	50.7 ± 13.7	51.9 ± 11.9
eGFR, mL/min/1.73m^2^	86.9 ± 12.9	77.9 ± 13.7	66.2 ± 18.1
Serum triglycerides ≥ 150 mg/dL, n (%)	0 (0%)	277 (18.9%)	45 (20.0%)
Low High Density Lipoprotein cholesterol^1^, n (%)	150 (34.9%)	575 (39.2%)	92 (40.8%)
Fasting blood glucose concentration ≥ 100 mg/dL, n (%)	10 (2.3%)	387 (26.4%)	100 (44.4%)
**Medical history**			
Chronic Kidney Disease stage^2^, n (%)			
1	171 (39.8%)	281 (19.1%)	14 (6.2%)
2	0(0%)	456 (31.1%)	75 (33.3%)
3	0(0%)	119 (8.1%)	56 (24.8%)
4	0(0%)	0(0%)	8 (3.5%)
5	0(0%)	0(0%)	2 (0.8%)
PREVENT equation 10-year total CVD risk %	1.9 ± 2.24	6.3 ± 5.0	19.1 ± 10.3
**Medication use**, n (%)			
Antihypertensive medications	0(0%)	718 (49.04%)	181 (80.4%)
Glucose lowering medications	0(0%)	146 (9.9%)	71 (31.5%)
Lipid lowering medications	14 (3.26%)	160 (10.9%)	41 (18.2%)

Data are presented as mean ± standard deviation or n (%).

1. Low High-Density Lipoprotein: <40 mg/dL for men and <50 mg/dL for women.

2. Chronic Kidney Disease stages: Stage 1= eGFR >= 90 mL mL/min/1.73m^2^ with albuminuria urine albumin to urine creatinine ratio based on spot or 24-hour urine values - ACR > 30 mg/g); Stage 2 = eGFR: 60–89 mL/min/1.73m^2^ with albuminuria; Stage 3 = eGFR 30–59 mL/min/1.73m^2^; Stage 4 = eGFR 14–29 mL/min/1.73m^2^; Stage 5 = eGFR <15 mL/min/1.73m^2^.

*eGFR* estimated glomerular filtration rate; *PREVENT equation* American Heart Association Predicting Risk of Cardiovascular Disease Events equation, *CVD* cardiovascular disease.

### Common metabolic risk factors in JHS adults with stage 2 and 3 CKM

All participants with stage 2 and stage 3 CKM had obesity, abdominal obesity or dysfunctional adipose tissue (Table 2). Hypertension was the most common metabolic risk factor among participants with stage 2 and stage 3 CKM. The three most common metabolic risk factor combinations among participants with stage 2 CKM, whether as single conditions or in combination with other conditions, were: 1) hypertension alone, 2) hypertension with moderate-high risk CKD, and 3) hypertension with metabolic syndrome (Figure S4). Among participants with stage 3 CKM, the three most common metabolic risk factor combinations, whether as single conditions or in combination with other conditions, were: 1) hypertension alone, 2) hypertension with moderate-high risk CKD, metabolic syndrome, and diabetes, and 3) hypertension with moderate-high risk CKD, and metabolic syndrome (Figure S5).

**Table 2:** Prevalence of obesity/overweight, abdominal obesity, dysfunctional adipose tissue, metabolic risk factors, and subclinical cardiovascular disease among Jackson Heart Study participants with stage 2 and 3 Cardiovascular-Kidney-Metabolic (CKM) syndrome at baseline.

	Cardiovascular-Kidney-Metabolic syndrome stage	
	Stage 2 (N = 1464)	Stage 3 (N = 225)
**Metabolic Risk Factors, n (%)**		
**Overweight/obesity, abdominal obesity, dysfunctional adipose tissue**		
Obesity	1399 (95.5%)	210 (93.3%)
Abdominal obesity	1010 (68.9%)	180 (80.0%)
Dysfunctional adipose tissue	704 (48.1%)	114 (50.7%)
Obesity, abdominal obesity, or dysfunctional adipose tissue	1464 (100%)	225 (100%)
**Metabolic risk factors**		
Hypertension, %	1166 (79.6%)	214 (95.1%)
Diabetes, %	232 (15.8%)	99 (44.0%)
Hypertriglyceridemia, %	461 (31.4%)	72 (32.0%)
Moderate – High risk CKD	575 (39.2%)	131 (58.2%)
Metabolic syndrome	623 (42.5%)	131 (58.2%)
**Number of metabolic risk factors, n (%)**		
0	0 (0%)	0 (0%)
1	544 (37.1%)	40 (17.7%)
2	439 (29.9%)	45 (20.0%)
3	308 (21.0%)	62 (27.5%)
4	154 (10.5%)	59 (26.2%)
5	19 (1.3%)	19 (8.4%)
**Subclinical CVD, n (%)**		
Left ventricular hypertrophy	0 (0%)	117 (52.0%)
High risk CVD (PREVENT equation 10-year total CVD risk ≥ 20%)	0 (0%)	132 (58.6%)

Obesity: Body Mass Index ≥ 25 kg/m^2^.

Abdominal obesity: waist circumference ≥ 102 cm for men and ≥ 88 cm for women.

Dysfunctional adipose tissue: fasting blood glucose ≥ 100–124 mg/dL and/or Hemoglobin A1c (HbA1c) between 5.7% and 6.4%.

See Table S1 for the definitions of the metabolic risk factors.

*CKD* chronic kidney disease; *PREVENT equation* American Heart Association Predicting Risk of Cardiovascular Disease Events equation, *CVD* cardiovascular disease.

### Incidence rate of stage 4 CKM

The incidence rate of stage 4 CKM among participants with stage 1 CKM, stage 2 CKM and stage 3 CKM was 1.4 (95% confidence interval [CI]: 0.4, 2.4), 7.0 (95% CI: 5.8, 8.2), and 25.4 (95% CI: 19.1, 31.8) per 1000 person-years, respectively (Table 3). Each metabolic risk factor was associated with higher multivariable adjusted risk for stage 4 CKM compared to stage 1 CKM. For those with hypertension, the adjusted HR (95% CI) was 3.25 (1.56, 6.80) in stage 2 CKM and 5.11 (2.04 12.78) in stage 3 CKM. Participants with versus without diabetes, hypertriglyceridemia, metabolic syndrome and moderate to high-risk CKD in stage 2 and stage 3 CKM had higher adjusted HR for developing stage 4 CKM.

**Table 3:** Incidence Rates and Hazard Ratios for Stage 4 Cardiovascular-Kidney-Metabolic (CKM) syndrome among Jackson Heart Study participants with Stage 1, 2 and 3 CKM syndrome and Associated Risk Factors.

	Number of participants	Number of participants developing Stage 4 CKM	Person-years of follow up	Incidence rate per 1000/person years (95% CI)	Hazard ratio (95% CI) compared to participants with stage 1 CKM		
CKM stage 1					Model 1	Model 2	Model 3
Overall	429	8	4915	1.6 (0.4, 2.7)	Reference	Reference	Reference
CKM stage 2							
Overall	1464	133	15737	8.4 (7.0, 9.8)	3.31 (1.60, 6.85)	3.23 (1.56, 6.68)	3.04 (1.47, 6.29)
Hypertension	1166	113	12445	9.0 (7.4, 10.7)	1.29 (0.80, 2.08)	1.35 (0.84, 2.19)	1.31 (0.87, 2.12)
Diabetes	232	43	2362	18.2 (12.7, 23.6)	2.47 (1.71, 3.57)	2.68 (1.85, 3.89)	2.56 (1.75, 3.75)
Hypertriglyceridemia	461	57	4834	11.7 (8.7, 14.8)	1.69 (1.19, 2.38)	1.63 (1.15, 2.30)	1.63 (1.15, 2.31)
Metabolic Syndrome	623	80	6511	12.2 (9.5, 14.9)	2.19 (1.54, 3.11)	2.24 (1.57, 3.18)	2.14 (1.49, 3.08)
Moderate – High risk CKD	575	56	6177	9.0 (6.6, 11.4)	0.92 (0.65, 1.31)	0.88 (0.62, 1.25)	0.88 (0.62, 1.25)
CKM stage 3							
Overall	225	62	1897	32.6 (24.5, 40.8)	6.28 (2.63, 15.03)	5.89 (2.46, 14.10)	4.86 (1.97, 12.00)
Hypertension	214	61	1784	34.1 (25.8, 42.7)	2.59 (0.35, 18.91)	2.80 (0.38, 20.60)	2.92 (0.39, 21.44)
Uncontrolled treated hypertension	127	39	1011	38.5 (26.4, 50.6)	1.25 (0.74, 2.11)	1.22 (0.71, 2.08)	1.25 (0.73, 2.13)
Uncontrolled untreated hypertension	33	5	308	16.2 (2.0, 30.4)	0.53 (0.21, 1.33)	0.54 (0.21, 1.40)	0.54 (0.21, 1.38)
Untreated hypertension	101	26	838	31.0 (19.0, 42.9)	0.84 (0.50, 1.39)	0.83 (0.50, 1.38)	0.84 (0.50, 1.39)
Controlled treated hypertension	129	47	996	47.1 (33.6, 60.6)	2.28 (1.24, 4.22)	2.30 (1.24, 4.24)	2.27 (1.23, 4.20)
Diabetes	99	37	752	49.1 (33.3, 64.9)	2.00 (1.20, 3.34)	1.96 (1.17, 3.28)	1.98 (1.18, 3.32)
Hypertriglyceridemia	72	18	574	31.3 (16.8, 45.7)	1.15 (0.66, 2.01)	1.16 (0.66, 2.02)	1.20 (0.68, 2.10)
Metabolic Syndrome	131	40	1073	37.2 (25.7, 48.8)	1.52 (0.89, 2.59)	1.52 (0.89, 2.58)	1.45 (0.84, 2.48)
Moderate – High risk CKD	131	38	1069	35.5 (24.2, 46.8)	1.02 (0.61, 1.72)	1.04 (0.62, 1.75)	1.02 (0.61, 1.72)

See Table S1 for the definitions of the metabolic risk factors.

Model 1: age, sex, education.

Model 2: Model 1 + smoking and alcohol use.

Model 3: Model 2 + body mass index.

*CKM* cardiovascular-kidney-metabolic syndrome, *CKD* chronic kidney disease.

### Incidence of stage 4 CKM by hypertension status in stage 2 and stage 3 CKM

Among 1464 JHS participants with stage 2 CKM, 828 (56.5%) had hypertension and other metabolic risk factors, 338 (23.1%) had hypertension with no other metabolic risk factors, and 298 (20.4%) did not have hypertension but had other metabolic risk factors (Table 4). The incidence rate (95% CI) per 1000 person years was 8.8 (7.0, 10.60) for hypertension and other metabolic risk factors, 5.1 (2,83, 7.26) for other metabolic risk factors without hypertension and 4.3 (2.4, 6.3) for hypertension alone. The adjusted HR for stage 4 CKM associated with having hypertension and other metabolic risk factors versus having other metabolic risk factors without hypertension for participants with stage 2 CKM was 1.45 (95% CI: 0.89, 2.37).

**Table 4:** Incident Rates and Hazard ratios for Stage 4 Cardiovascular-Kidney-Metabolic (CKM) syndrome among Jackson Heart Study participants with Stage 2 and Stage 3 with and without hypertension.

	Number of participants	Number of participants developing incident Stage 4 CKM	Person-years of follow up	Incidence rate per 1000/person years (95% CI)	Hazard ratio (95% CI)		
					Model 1	Model 2	Model 3
CKM Stage 2^1^ (N = 1464)							
No hypertension with other risk factors	298	20 (15.0%)	3291	6.0 (3.4, 8.7)	reference	reference	reference
Hypertension and other risk factors	828	94 (70.6%)	8741	10.7 (8.5, 12.9)	1.49 (0.91, 2.42)	1.54 (0.94, 2.50)	1.48 (0.90, 2.41)
Hypertension only	338	19 (14.3%)	3703	5.1 (2.8, 7.4)	0.80 (0.42, 1.49)	0.86 (0.46, 1.62)	0.86 (0.46, 1.63)
CKM Stage 3^2^ (N = 225)							
No hypertension and have other risk factors	11	1 (1.6%)	112	8.8 (−8.5, 26.2)	Not Assessed^a^		
Hypertension and other risk factors	183	57 (91.9%)	1493	38.1 (28.2, 48.0)			
Hypertension only	31	4 (6.4%)	291	13.7 (0.2, 27.1)			

Model 1: age, sex, education.

Model 2: Model 1 + smoking and alcohol use.

Model 3: Model 2 + body mass index.

1. For participants with Stage 2 CKM and with no hypertension and with other metabolic risk factors (n = 298): 206 (69%) had 1 risk factor, 62 (21%) had 2 risk factors, 27 (9%) had 3 risk factors, and 3 (1%) had 4 risk factors. For participants with Stage 2 CKM with hypertension and with other metabolic risk factors (n = 828): 377 (46%) had 2 risk factors, 281 (34%) had 3 risk factors, 151 (18%) had 4 risk factors, and 19 (2%) had all 5 risk factors.

2. For participants with Stage 3 CKM with no hypertension and with other metabolic risk factors (n = 11): 9 (82%) had 1 risk factor, 1 (9%) had 2 risk factors and 1 (9%) had 3 risk factors.

For participants with Stage 3 CKM with hypertension and with other metabolic risk factors (n = 183): 44 (24%) had 2 risk factors, 61 (33%) had 3 risk factors, 59 (32%) had 4 risk factors, and 19 (11%) had all 5 risk factors.

*CKM* cardiovascular-kidney-metabolic syndrome; *CI* confidence interval.

^a^We did not fit the Cox regression model given the small sample size and event size among participants with stage 3 CKM without hypertension and with other risk factors.

Among participants with stage 3 CKM, 95% (214/225 participants) had hypertension. Moreover, among participants with stage 3 CKM, 1 out of 11 participants who did not have hypertension but had other metabolic risk factors developed stage 4 CKM compared to 56 out of 183 participants who had hypertension and other metabolic risk factors and 4 out of 31 participants who had hypertension with no other metabolic risk factors that developed stage 4 CKM.

### Risk of stage 4 CKM by each metabolic risk factor stage 2 and stage 3 CKM

The adjusted HR for incident stage 4 CKM associated with having versus not having hypertension was 1.30 (95% CI: 0.80, 2.11) and 2.82 (95% CI: 0.41, 22.29) for participants with stage 2 and stage 3 CKM, respectively (Table S2). Having versus not having diabetes was associated with an increased risk for developing stage 4 CKM for participants with stage 2 and stage 3 CKM. Hypertriglyceridemia and metabolic syndrome were associated with incident stage 4 CKM among participants with stage 2 CKM but not stage 3 CKM. Moderate to high-risk CKD was not associated with incident stage 4 CKM in either stage.

## Discussion

This study has several important findings for public health and clinical practice. Hypertension was the most common metabolic risk factor among participants with stage 2 and stage 3 CKM and was associated with a higher risk of progression to stage 4 CKM compared to participants with stage 1 CKM. Most participants with stage 2 and 3 CKM had two or more metabolic risk factors. Among participants with other metabolic risk factors, those with coexisting hypertension had higher incidence rate of stage 4 CKM.

The AHA Presidential Advisory on CKM syndrome defined, staged, and provided algorithms for its prevention and treatment [1]. According to NHANES data from 2011 to 2018, a substantial proportion of Black adults in the US have stage 2 CKM [18]. Furthermore, hypertension was the most common metabolic risk factor among US adults with stage 2 CKM [19]. In another analysis using NHANES data from 2011 to 2020, 50.5 and 7.5% of Black US adults, 20 years of age and older, had stage 2 and 3 CKM, respectively [2]. The findings from the current study confirm the high prevalence of stage 2 CKM and hypertension in Black adults and extend prior work by demonstrating the association of hypertension with an increased risk for progression to stage 4 CKM.

A higher percentage of CVD cases has been attributed to hypertension compared with any other modifiable risk factor [20, 21]. It has been estimated that 33% of CVD events among Black US adults are attributed to hypertension [21]. The high prevalence of hypertension among Black adults and the large proportion of CVD events that could be attributed to hypertension emphasizes the importance of hypertension prevention. Moreover, in the current analysis, Black adults with stage 2 and stage 3 CKM and hypertension had an increased risk of developing stage 4 CKM compared to those with stage 1 CKM. These findings suggest that public health interventions aimed at preventing hypertension may help slow CKM progression, which includes reducing the incidence of CVD among Black adults [22, 23].

Randomized trials have identified several non-pharmacological approaches for lowering BP and preventing hypertension, including promoting a heart-healthy lifestyle, encouraging physical activity, adopting a healthy diet, reducing dietary salt consumption, maintaining a healthy weight, avoiding excessive drinking, and getting sufficient sleep [24, 25]. Given that all participants with stage 2 and stage 3 CKM in the current study had obesity, abdominal obesity, or dysfunctional adipose tissue, weight loss counseling and lifestyle modification may be effective therapies to prevent the development of hypertension [26]. A systematic review of eight trials showed that behavioral weight loss interventions reduced SBP by 4.5 mmHg and DBP by 3.2 mmHg [27]. In addition to non-pharmacological interventions, a randomized trial of adults who were obese without diabetes demonstrated that tirzepatide, a dual agonist for glucagon-like peptide-1 receptor (GLP-1) and glucose-dependent insulinotropic polypeptide (GIP), reduced SBP by 7.4 mmHg for 5 mg daily dose and by 10.6 mmHg at a 10 mg daily dose compared to placebo over 72 weeks [28, 29]. While weight loss interventions reduce BP, their role in preventing the progression of CKM by lowering BP has not been directly shown. Building on this evidence, our findings suggest that the rate of CKM progression is high among individuals with hypertension, highlighting it as a key risk factor where targeted interventions should be directed. Prior studies have demonstrated the effectiveness of culturally tailored programs including pharmacist-led BP management, faith-based initiatives, and barbershop interventions in prevention of hypertension and improving BP control among Black populations [30, 31]. These approaches also align with public health recommendations to improve outreach, overcome mistrust, and address barriers to care that disproportionately affect Black adults [32, 33]. These strategies may help reduce CKM progression and cardiovascular risk among Black adults by targeting the most prevalent and clustered modifiable risk factors.

The increasing prevalence of multimorbidity among US adults adds complexity to hypertension management and prevention efforts by requiring individualized treatment plans, complicating medication regimens, and increasing the risk of adverse events and care fragmentation [34]. The prevalence of multiple chronic conditions among US adults with hypertension has increased substantially, rising by over 10% from 1999–2000 to 2017–2020 [34]. In 2017–2020, 52% of US adults with hypertension had three or more additional chronic conditions. In the current analysis, most adults in JHS with stages 2 and 3 CKM had two or more metabolic risk factors, with hypertension being the most common in both stages. Also, participants with hypertension and other metabolic risk factors in the current study had higher incidence of clinical CVD compared to those with other metabolic risk factors but not hypertension. Having multiple chronic conditions has been associated with increased mortality, worse quality of life, longer hospital stays, increased barriers to medication adherence and high medication cost [35, 36]. This underscores the potential benefit of preventing the development of multiple metabolic risk factors and emphasizes a comprehensive approach to hypertension prevention within the context of other risk factors.

The study has several strengths. JHS enrolled a large sample size of Black US adults. Data in the JHS were collected following standardized protocols. The current analysis has potential limitations. Only a sample of JHS participants completed the echocardiogram at baseline and echocardiograms were not conducted at visits 2 and 3. Therefore, we were unable to study the transition from stage 1 or stage 2 CKM to stage 3 CKM. Additionally, given the moderate sample size we did not assess the role of hypertension in CKM syndrome by sex. Furthermore, we lacked data on female-specific risk factors, such as autoimmune diseases and polycystic ovary syndrome, which limited our ability to evaluate their contribution to CKM syndrome. We were unable to calculate the population attributable risk for hypertension due to the overlap between hypertension and other metabolic risk factors among participants with both stage 2 and stage 3 CKD. While the JHS began over two decades ago, structural inequities in preventive care and hypertension management persist today, and racial disparities in cardiovascular outcomes persist [32, 33, 37]. Therefore, the current study’s findings remain relevant for understanding CKM progression in a historically underrepresented and high-risk population. However, the cohort was drawn from a single geographic region, Jackson, Mississippi, which may limit generalizability to Black adults in other U.S. settings.

In this study of Black adults from the JHS, hypertension was the most common metabolic risk factor among participants with CKM stages 2 and 3. In addition, hypertension was associated with a higher risk for developing stage 4 CKM compared to those with stage 1 CKM. These findings emphasize the relevance of hypertension within the CKM framework.

## Summary

### What is known about topic

- Cardiovascular kidney metabolic syndrome (CKM) is characterized by the interaction of metabolic risk factors, chronic kidney disease and cardiovascular disease (CVD) which leads to multi-organ dysfunction and adverse cardiovascular outcomes.
- CKM is defined by 4 progressive mutually exclusive stages.
- Hypertension is a common metabolic risk factor among United States adults, and its prevalence is a higher among adults with versus without obesity.

### What this study adds

- This study highlights hypertension as the most common metabolic risk factor in Black adults with stage 2 and 3 CKM and its association with progression to stage 4 CKM (having a CVD event).
- It extends prior National Health and Nutrition Examination Survey findings by demonstrating how hypertension increases the risk of developing clinical CVD among Black Adults with stage 2 and 3 CKM.

## Supplementary information

Supplementary material

## Data Availability

Deidentified participant data from the Jackson Heart Study can be requested from the study’s coordinating center with an approved manuscript proposal and Data and Materials Distribution Agreement. In addition, Jackson Heart Study can be obtained from the Biologic Specimen and Data Repository Information Coordinating Center of the National Heart, Lung, and Blood Institute.

## References

[1] Ndumele CE, Rangaswami J, Chow SL, Neeland IJ, Tuttle KR, Khan SS, et al. Cardiovascular-kidney-metabolic health: a presidential advisory from the American Heart Association. Circulation. 2023;148:1606–35. 37807924 10.1161/CIR.0000000000001184

[2] Aggarwal R, Ostrominski JW, Vaduganathan M Prevalence of cardiovascular-kidney-metabolic syndrome stages in US adults, 2011–2020. JAMA 2024. 10.1001/jama.2024.6892. 10.1001/jama.2024.6892 PMC11079779 38717747

[3] Muntner P, Carey RM, Gidding S, Jones DW, Taler SJ, Wright JT, et al. Potential US Population Impact of the 2017 ACC/AHA High Blood Pressure Guideline. Circulation. 2018;137:109–18. 29133599 10.1161/CIRCULATIONAHA.117.032582 PMC5873602

[4] Jones DW, Ferdinand KC, Taler SJ, Johnson HM, Shimbo D, Abdalla M, et al. 2025 AHA/ACC/AANP/AAPA/ABC/ACCP/ACPM/AGS/AMA/ASPC/NMA/PCNA/SGIM Guideline for the prevention, detection, evaluation and management of high blood pressure in adults: a report of the American College of Cardiology/American Heart Association Joint Committee on Clinical Practice Guidelines. Circulation; **0**, 10.1161/CIR.0000000000001356. 10.1161/CIR.0000000000001356 40811497

[5] Virani SS, Alonso A, Benjamin EJ, Bittencourt MS, Callaway CW, Carson AP, et al. Heart disease and stroke statistics-2020 update: a report from the American Heart Association. Circulation. 2020;141:e139–e596. 31992061 10.1161/CIR.0000000000000757

[6] Taylor HA, Wilson JG, Jones DW, Sarpong DF, Srinivasan A, Garrison RJ, et al. Toward resolution of cardiovascular health disparities in African Americans: design and methods of the Jackson Heart Study. Ethn Dis. 2005;15:S6-4–17. 16320381

[7] Carpenter MA, Crow R, Steffes M, Rock W, Heilbraun J, Evans G, et al. Laboratory, reading center, and coordinating center data management methods in the Jackson Heart Study. Am J Med Sci. 2004;328:131–44. 15367870 10.1097/00000441-200409000-00001

[8] Seals SR, Colantonio LD, Tingle JV, Shimbo D, Correa A, Griswold ME, et al. Calibration of blood pressure measurements in the Jackson Heart Study. Blood Press Monit. 2019;24:130–6. 30998553 10.1097/MBP.0000000000000379 PMC7225013

[9] Wilkins JT, Ning H, Berry J, Zhao L, Dyer AR, Lloyd-Jones DM. Lifetime risk and years lived free of total cardiovascular disease. JAMA. 2012;308:1795–801. 23117780 10.1001/jama.2012.14312 PMC3748966

[10] Delgado C, Baweja M, Crews DC, Eneanya ND, Gadegbeku CA, Inker LA, et al. A unifying approach for GFR estimation: recommendations of the NKF-ASN task force on reassessing the inclusion of race in diagnosing kidney disease. Am J Kidney Dis. 2022;79:268–288.e1. 34563581 10.1053/j.ajkd.2021.08.003

[11] Wang W, Young BA, Fülöp T, de Boer IH, Boulware LE, Katz R, et al. Effects of serum creatinine calibration on estimated renal function in African Americans: the Jackson Heart Study. Am J Med Sci. 2015;349:379–84. 25806862 10.1097/MAJ.0000000000000446 PMC4414728

[12] Patel VG, Gupta DK, Terry JG, Kabagambe EK, Wang TJ, Correa A, et al. Left Ventricular function across the spectrum of body mass index in African Americans: The Jackson Heart Study. JACC Heart Fail. 2017;5:182–90. 28254124 10.1016/j.jchf.2016.12.020 PMC5338642

[13] Kamimura D, Loprinzi PD, Wang W, Suzuki T, Butler KR, Mosley TH, et al. Physical activity is associated with reduced left ventricular mass in obese and hypertensive African Americans. Am J Hypertens. 2017;30:617–23. 28369190 10.1093/ajh/hpx044 PMC5861530

[14] Lang RM, Badano LP, Mor-Avi V, Afilalo J, Armstrong A, Ernande L, et al. Recommendations for cardiac chamber quantification by echocardiography in adults: an update from the American Society of Echocardiography and the European Association of Cardiovascular Imaging. J Am Soc Echocardiogr. 2015;28:1–39.e14. 25559473 10.1016/j.echo.2014.10.003

[15] Keku E, Rosamond W, Taylor HA, Garrison R, Wyatt SB, Richard M, et al. Cardiovascular disease event classification in the Jackson Heart Study: methods and procedures. Ethn Dis. 2005;15:S6-62–70. 16317987

[16] Koo P, Gjelsvik A, Choudhary G, Wu W-C, Wang W, McCool FD, et al. Prospective association of physical activity and heart failure hospitalizations among black adults with normal ejection fraction: The Jackson Heart Study. J Am Heart Assoc. 2017;6:e006107. 28882818 10.1161/JAHA.117.006107 PMC5634276

[17] Spahillari A, Talegawkar S, Correa A, Carr JJ, Terry JG, Lima J, et al. Ideal cardiovascular health, cardiovascular remodeling, and heart failure in blacks: The Jackson Heart Study. Circ Heart Fail. 2017;10:e003682. 28209767 10.1161/CIRCHEARTFAILURE.116.003682 PMC5319800

[18] Minhas AMK, Mathew RO, Sperling LS, Nambi V, Virani SS, Navaneethan SD, et al. Prevalence of the cardiovascular-kidney-metabolic syndrome in the United States. J Am Coll Cardiol. 2024;83:1824–6. 38583160 10.1016/j.jacc.2024.03.368

[19] Tian Z, Soltani S, Bauersachs J, Schmidt-Ott K, Melk A, Schmidt BM. High Prevalence of the Cardiovascular-Kidney-Metabolic Syndrome Among US Adults From 1999–2020 - An analysis of the NHANES survey. 2024: 2024.03.04.24303751. 10.1016/j.xkme.2025.101234 PMC12874573 41659825

[20] Lewington S, Clarke R, Qizilbash N, Peto R, Collins R, Prospective Studies Collaboration. Age-specific relevance of usual blood pressure to vascular mortality: a meta-analysis of individual data for one million adults in 61 prospective studies. Lancet. 2002;360:1903–13. 12493255 10.1016/s0140-6736(02)11911-8

[21] Clark D, Colantonio LD, Min Y-I, Hall ME, Zhao H, Mentz RJ, et al. Population-attributable risk for cardiovascular disease associated with hypertension in black adults. JAMA Cardiol. 2019;4:1194–202. 31642869 10.1001/jamacardio.2019.3773 PMC6813577

[22] Bryant KB, Moran AE, Kazi DS, Zhang Y, Penko J, Ruiz-Negrón N, et al. Cost-effectiveness of Hypertension Treatment by Pharmacists in Black Barbershops. Circulation. 2021;143:2384–94. 33855861 10.1161/CIRCULATIONAHA.120.051683 PMC8206005

[23] Schoenthaler AM, Lancaster KJ, Chaplin W, Butler M, Forsyth J, Ogedegbe G. Cluster randomized clinical trial of FAITH (Faith-Based Approaches in the Treatment of Hypertension) in blacks. Circ Cardiovasc Qual Outcomes. 2018;11:e004691. 30354579 10.1161/CIRCOUTCOMES.118.004691

[24] 2017 ACC/AHA/AAPA/ABC/ACPM/AGS/APhA/ASH/ASPC/NMA/PCNA Guideline for the Prevention, Detection, Evaluation, and Management of High Blood Pressure in Adults: A Report of the American College of Cardiology/American Heart Association Task Force on Clinical Practice Guidelines | Hypertension. https://www.ahajournals.org/doi/10.1161/HYP.0000000000000065 (accessed 20 Oct 2022).

[25] Lloyd-Jones DM, Allen NB, Anderson CAM, Black T, Brewer LC, Foraker RE, et al. Life’s Essential 8: Updating and Enhancing the American Heart Association’s construct of cardiovascular health: a presidential advisory from the American Heart Association. Circulation. 2022;146:e18–e43. 35766027 10.1161/CIR.0000000000001078 PMC10503546

[26] Ndumele CE, Neeland IJ, Tuttle KR, Chow SL, Mathew RO, Khan SS, et al. A Synopsis of the evidence for the science and clinical management of cardiovascular-kidney-metabolic (CKM) syndrome: a scientific statement from the American Heart Association. Circulation. 2023;148:1636–64. 37807920 10.1161/CIR.0000000000001186

[27] Semlitsch T, Krenn C, Jeitler K, Berghold A, Horvath K, Siebenhofer A. Long-term effects of weight-reducing diets in people with hypertension. Cochrane Database Syst Rev. 2021;2:CD008274. 33555049 10.1002/14651858.CD008274.pub4 PMC8093137

[28] Jastreboff AM, Aronne LJ, Ahmad NN, Wharton S, Connery L, Alves B, et al. Tirzepatide once weekly for the treatment of obesity. N Engl J Med. 2022;387:205–16. 35658024 10.1056/NEJMoa2206038

[29] de Lemos JA, Linetzky B, le Roux CW, Laffin LJ, Vongpatanasin W, Fan L, et al. Tirzepatide reduces 24-Hour ambulatory blood pressure in adults with body mass index ≥27 kg/m2: SURMOUNT-1 ambulatory blood pressure monitoring substudy. Hypertension. 2024;81:e41–e43. 38314555 10.1161/HYPERTENSIONAHA.123.22022 PMC10956672

[30] Ogedegbe G, Chaplin W, Schoenthaler A, Statman D, Berger D, Richardson T, et al. A practice-based trial of motivational interviewing and adherence in hypertensive African Americans. Am J Hypertens. 2008;21:1137–43. 18654123 10.1038/ajh.2008.240 PMC3747638

[31] Victor RG, Lynch K, Li N, Blyler C, Muhammad E, Handler J, et al. A cluster-randomized trial of blood-pressure reduction in black barbershops. N Engl J Med. 2018;378:1291–301. 29527973 10.1056/NEJMoa1717250 PMC6018053

[32] Ferdinand DP, Nedunchezhian S, Ferdinand KC. Hypertension in African Americans: Advances in community outreach and public health approaches. Prog Cardiovasc Dis. 2020;63:40–45. 31863786 10.1016/j.pcad.2019.12.005

[33] Abrahamowicz AA, Ebinger J, Whelton SP, Commodore-Mensah Y, Yang E. Racial and ethnic disparities in hypertension: barriers and opportunities to improve blood pressure control. Curr Cardiol Rep. 2023;25:17–27. 36622491 10.1007/s11886-022-01826-x PMC9838393

[34] Alanaeme CJ, Ghazi L, Akinyelure OP, Wen Y, Christenson A, Poudel B, et al. Trends in the prevalence of multiple chronic conditions among US adults with hypertension from 1999–2000 through 2017–2020. Am J Hypertens. 2024;37:493–502. 38576398 10.1093/ajh/hpae040 PMC11519032

[35] Fortin M, Soubhi H, Hudon C, Bayliss EA, van den Akker M. Multimorbidity’s many challenges. BMJ. 2007;334:1016–7. 17510108 10.1136/bmj.39201.463819.2C PMC1871747

[36] Peacock E, Krousel-Wood M. Adherence to antihypertensive therapy. Med Clin North Am. 2017;101:229–45. 27884232 10.1016/j.mcna.2016.08.005 PMC5156530

[37] Products - Data Briefs - Number 289 - October 2017. 2019. https://www.cdc.gov/nchs/products/databriefs/db289.htm (accessed 11 Aug 2025).

