# Supplementary material for: Role of hypertension in the cardiovascular-kidney-metabolic syndrome among black adults: The Jackson Heart Study
Source: J Hum Hypertens. 2025 Oct 4;39(12):822–30. doi: 10.1038/s41371-025-01078-6 (PMC12685740; doi:10.1038/s41371-025-01078-6)
Supplement: Supplementary file 1 — Supplementary material [file 41371_2025_1078_MOESM1_ESM.docx]

**SUPPLEMENTARY MATERIALS**

**Role of Hypertension in the Cardiovascular-Kidney-Metabolic Syndrome among Black Adults: The Jackson Heart Study**

Lama Ghazi, MD PhD^1^, Medha Dubal, MPH^1^, Alain Bertoni, MD MPH^2^, April Carson, PhD^3^, Bessie A. Young MD MPH^4^, Cora E. Lewis, MD^1^, Chibuike J. Alanaeme, MSPH^1^, Dayna A. Johnson, PhD MPH^5^, Daichi Shimbo, MD^6^, Kathryn Foti, PhD MPH^1^, Lisandro D. Colantonio, MD PhD^1^, Milla Arabadjian, PhD RN^7^, Rikki Tanner, PhD MPH^1^, Paul Muntner, PhD^1,8^

1. Department of Epidemiology, School of Public Health, University of Alabama at Birmingham, Birmingham, AL, USA.
2. Wake Forest School of Medicine, Department of Epidemiology and Prevention, Winston-Salem, NC.
3. Department of Medicine, University of Mississippi Medical Center, Jackson, MS, USA.
4. Division of Nephrology, Department of Medicine, and Office of Healthcare Equity, University of Washington, Seattle, WA.
5. Department of Epidemiology, Emory University Rollins School of Public Health, Atlanta, Georgia.
6. The Columbia Hypertension Center and Lab, Columbia University Irving Medical Center, New York, New York, United Kingdom.
7. Center for Population and Health Services Research, Department of Foundations of Medicine, New York University Grossman Long Island School of Medicine, Mineola, NY.
8. Perisphere Real World Evidence, Austin, Texas.

Short title: Hypertension and CKM in Black adults

**Corresponding Author:**
Lama Ghazi, MD PhD
Department of Epidemiology, School of Public Health
University of Alabama at Birmingham
1665 University Blvd, Birmingham, AL 35233
Phone: 205-975-5792 | Email: [lghazi@uab.edu](mailto:lghazi@uab.edu)

**Table S1. Definition of Cardiovascular-Kidney-Metabolic Stages.**

| **CKM Stages** | **Definition** |
| --- | --- |
| Stage 0 | At baseline have:  Normal body mass index (BMI; < 25 kg/m^2^) and  Normal waist circumference (<102 cm for men and < 88 cm for women), and  Normoglycemia (fasting blood glucose < 100 mg/dL and Hemoglobin A1C (HbA1c) < 5.7%)  and  Did not have metabolic risk factors (see Stage 2) or chronic kidney disease (CKD)  and  Did not have sub-clinical cardiovascular disease (CVD)  and  Did not have clinical CVD |
| Stage 1 | At baseline, have one or more of the following three factors:  1. Being overweight/obese (BMI ≥ 25 kg/m^2^)  2. Having abdominal obesity (waist circumference ≥ 102 cm for men and ≥  88 cm for women) or  3. Having dysfunctional adipose tissue (fasting blood glucose between 100 and 124 mg/dL and/or HbA1c between 5.7% and 6.4%) at baseline visit  and    Did not have metabolic risk factors (see Stage 2) or CKD  and  Did not have clinical CVD |
| Stage 2^†^ | At baseline having one or more of the following five metabolic risk factor:   1. Hypertension: taking antihypertensive medication (self-reported) or having a systolic blood pressure (SBP) ≥ 130 mm Hg or having a diastolic blood pressure (DBP) ≥ 80 mm Hg. 2. Diabetes: having an HbA1c ≥ 6.5% or a fasting blood glucose ≥ 126 mg/dL or using glucose-lowering medication. 3. Hypertriglyceridemia was defined as having serum triglycerides ≥ 125 mg/dL. 4. Metabolic syndrome was defined based on the adult ATP-III criteria. If participants had 3 or more of the following 5 criteria they will be considered to have the metabolic syndrome. 5. High serum triglyceride concentration (≥ 150 mg/dL) 6. High density lipoprotein (HDL) cholesterol < 40 mg/dL for men and < 50 mg/dL for women 7. Waist circumference > 102 cm for men and > 88 cm for women 8. Fasting blood glucose concentration ≥ 100 mg/dL 9. SBP ≥ 130 mm Hg or DBP ≥ 80 mm Hg and/or using antihypertensive medication 10. Moderate to high-risk CKD was defined according to the Kidney Disease Improving Global Outcomes (KDIGO) criteria as having stage 2 CKD (estimated glomerular filtration rate [eGFR] 60-89 mL/min/1.73m^2^ with albuminuria among participants who had urine albumin to urine creatinine ratio [ACR] measured at baseline) or stage 3 CKD (eGFR 30-59 mL/min/1.73m^2^).   and  Did not have sub-clinical CVD  and  Did not have clinical CVD |
| Stage 3 | At baseline, having sub-clinical CVD, stage 4 or 5 CKD or high predicted 10-year risk for total CVD among participants who  Have overweight/obese or  Have abdominal obesity or  Have dysfunctional adipose tissue or  Have one or more metabolic risk factors.  *Sub-clinical CVD was defined as having left ventricular hypertrophy at the study baseline.*  *The 10-year CVD risk was estimated with the AHA Predicting Risk of CVD EVENTs (PREVENT) equations. High risk was defined as ≥ 20% 10-year CVD risk.* |
| Stage 4 | During follow up, having a CVD event including coronary heart disease (CHD), heart failure, or stroke among participants who   Have overweight/obese or  Have abdominal obesity or  Have dysfunctional adipose tissues or other metabolic risk factors. |

ACR: urine albumin to urine creatinine; BMI: Body Mass Index; CHD: coronary heart disease; CKD: Chronic Kidney Disease; DBP: diastolic blood pressure; eGFR: estimated glomerular filtration rate; HbA1c: Hemoglobin A1C; HDL: High Density Lipoprotein; KDIGO: Kidney Disease Improving Global Outcomes; PREVENT equation: AHA Predicting Risk of CVD EVENTs; SBP: Systolic Blood Pressure

† Stage 2 CKM did not require participants have obesity, abdominal obesity or dysfunctional adipose tissue. However, all JHS participants with one or more metabolic risk factor had obesity, abdominal obesity or dysfunctional adipose tissue.

**Table S2: Hazard ratios for incident stage 4 Cardiovascular-Kidney-Metabolic (CKM) syndrome for each metabolic risk factor among Jackson Heart Study participants with Stage 2 and Stage 3 CKM**

|  | Hazard ratio (95% confidence interval) | | |
| --- | --- | --- | --- |
|  | Model 1 | Model 2 | Model 3 |
| **Among participants with stage 2 CKM** | | | |
| Hypertension | 1.29 (0.80, 2.08) | 1.35 (0.83, 2.18) | 1.30 (0.80, 2.11) |
| Diabetes | 2.41 (1.66, 3.49) | 2.60 (1.79, 3.78) | 2.47 (1.68, 3.63) |
| Hypertriglyceridemia | 1. 64 (1.16, 2.33) | 1.59 (1.12, 2.25) | 1.59 (1.12, 2.25) |
| Metabolic Syndrome | 2.15 (1.51, 3.05) | 2.18 (1.53, 3.10) | 2.08 (1.44, 2.99) |
| Moderate – High risk CKD | 0.89 (0.62, 1.27) | 0.83 (0.59, 1.21) | 0.85 (0.59, 1.21) |
| **Among participants with stage 3 CKM** | | | |
| Hypertension | 2.68 (0.36, 19.53) | 2.88 (0.39, 21.12) | 2.82 (0.41, 22.29) |
| Diabetes | 1.92 (1.15, 3.22) | 1.89 (1.13, 3.18) | 1.90 (1.13, 3.20) |
| Hypertriglyceridemia | 1.06 (0.60, 1.88) | 1.07 (0.60, 1.89) | 1.11 (0.63, 1.98) |
| Metabolic Syndrome | 1.60 (0.93, 2.74) | 1.59 (0.93, 2.73) | 1.52 (0.88, 2.62) |
| Moderate – High risk CKD | 0.96 (0.57, 1.62) | 0.98 (0.58, 1.65) | 0.95 (0.56, 1.60) |

CKM: Cardiovascular-Kidney-Metabolic syndrome

See Table 1 for the definitions of the metabolic risk factors.

Model 1: age, sex, education

Model 2: Model 1 + smoking and alcohol use

Model 3: Model 2 + body mass index

**Figure S1. Flow chart of inclusion and exclusion criteria**

Jackson Heart study participants

N= 5306

After excluding participants who have CVD at baseline.

n= 4281

After excluding participants who are missing age and sex.

n=4281

After excluding participants who are missing education, smoking, alcohol status

n= 4212

After excluding participants missing SBP, DBP, or waist circumference

n= 4191

After excluding participants missing HbA1c, fasting blood glucose, serum triglyceride, total cholesterol, HDL cholesterol, serum creatinine

n= 3795

After excluding participants missing information on antihypertensive medication, glucose lowering medication, lipid lowering medications

n= 3728

After excluding participants missing incident CHD, incident HF, or incident stroke

n= 3572

After excluding participants missing left ventricular mass index

n= 2385

After excluding participants with CKM stage 0

n=2123

After excluding participants who did not fit the criteria of stage 1, 2 or 3 CKM *

n= 2118

CVD: Cardiovascular Disease, SBP: Systolic Blood Pressure, DBP: Diastolic Blood Pressure, HbA1c: Hemoglobin A1c; HDL: High density lipoprotein; CHD: Chronic Heart Disease, LVH: Left Ventricular Hypertrophy; CKM: Cardiovascular-Kidney-Metabolic.

* These participants had subclinical CVD but were neither obese nor overweight, did not have abdominal obesity or dysfunctional adipose tissue, and had no metabolic risk factors. Please refer to Table 1 for the definitions of CKM stages. Please refer to Table 1 for the definitions of CKM stages.

**Figure S2. Histogram plots for systolic blood pressure among Jackson Heart Study participants with Cardiovascular-Kidney-Metabolic (CKM) syndrome stage 1, 2 and 3.**


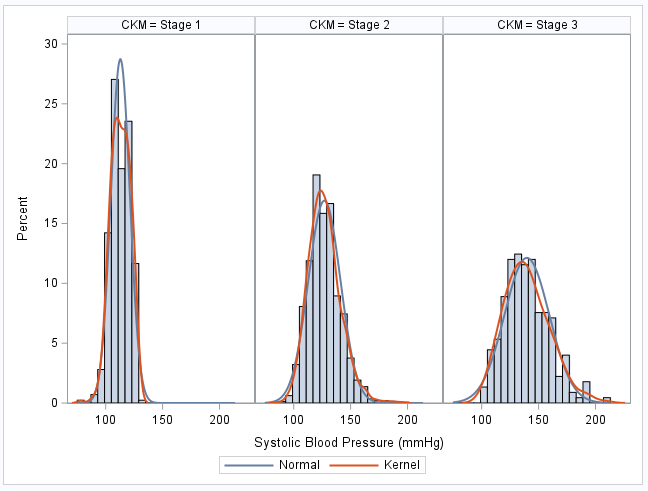


Mean systolic blood pressure (95% confidence interval):

- Stage 1: 113 (113.1, 113.7)
- Stage 2: 127 (126.2, 127.7)
- Stage 3: 140 (137.1, 142.3)

**Figure S3. Histogram plots for diastolic blood pressure among Jackson Heart Study participants with Cardiovascular-Kidney-Metabolic (CKM) syndrome stage 1, 2 and 3.**


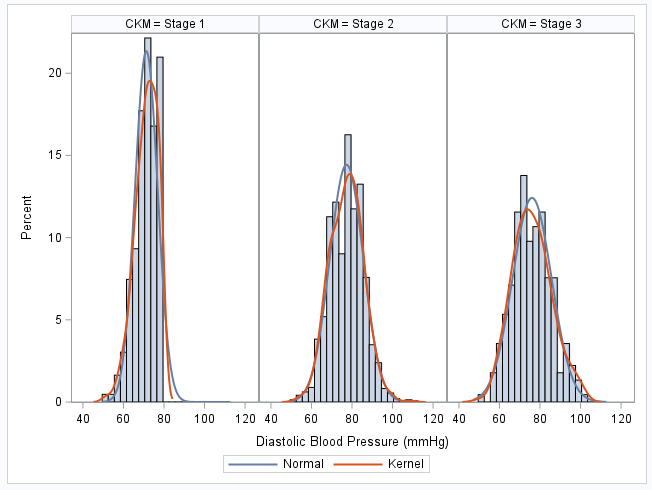


Mean diastolic blood pressure (confidence interval):

- Stage 1: 71 (70.8, 71.8)
- Stage 2: 77 (76.9, 77.7)
- Stage 3: 76 (74.8, 77.3)

**Figure S4. Prevalence of different combinations of metabolic risk factors among Jackson Heart Study participants with Cardiovascular-Kidney-Metabolic (CKM) syndrome stage 2**


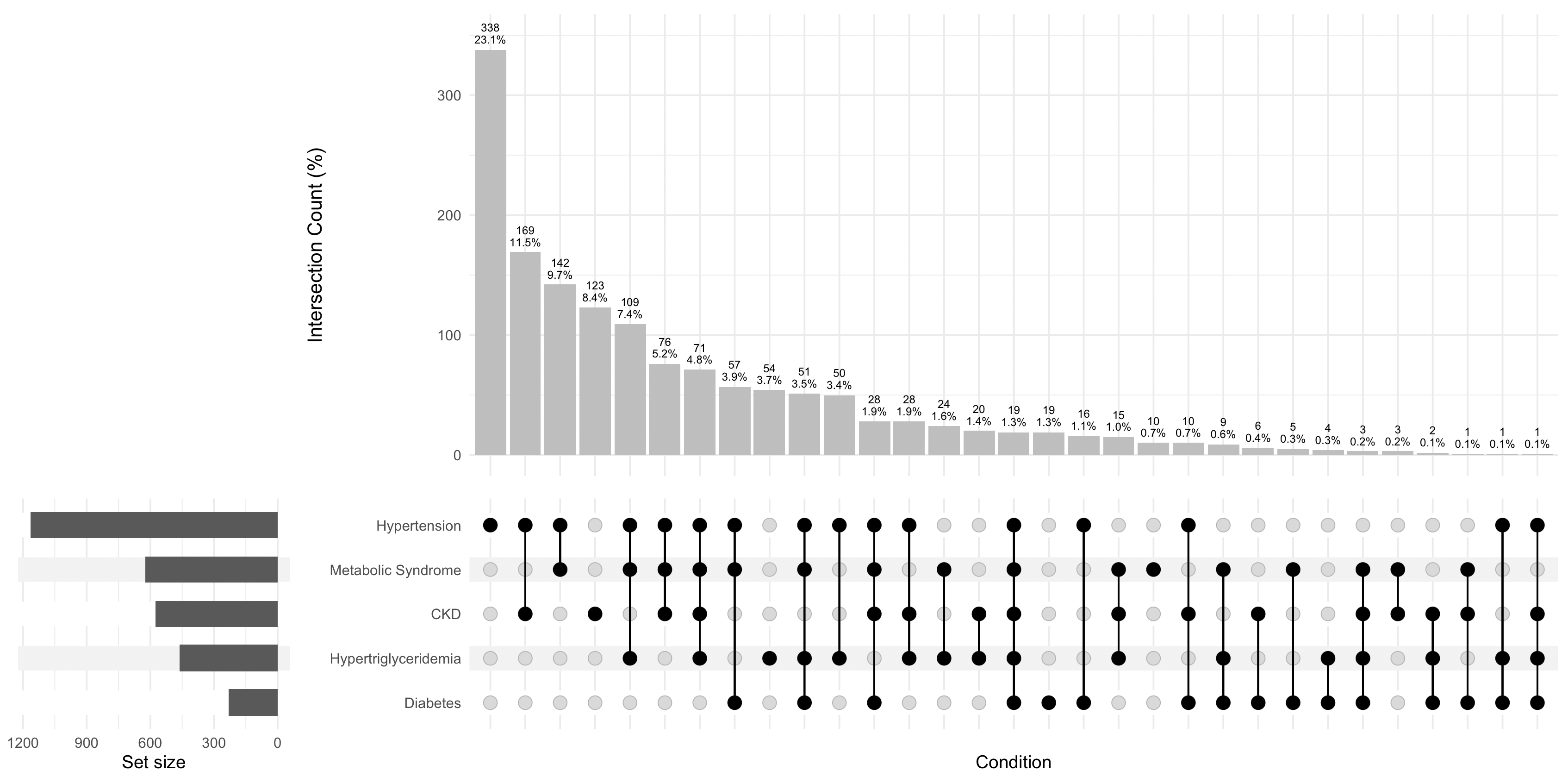


Hypertension: taking antihypertensive medication or having a systolic blood pressure ≥ 130 mm Hg or having a diastolic blood pressure ≥ 80 mm Hg
Diabetes: HbA1c ≥ 6.5% or a fasting blood glucose ≥ 126 mg/dL or using glucose-lowering medication.
Hypertriglyceridemia: serum triglycerides ≥ 125 mg/dL
Metabolic syndrome: If participants had 3 or more of the following 5 criteria they were considered to have the metabolic syndrome. 1) High serum triglyceride concentration (≥ 150 mg/dL); 2) High density lipoprotein (HDL) cholesterol < 40 mg/dL for men and < 50 mg/dL for women; 3) Waist circumference > 102 cm for men and > 88 cm for women; 4) Fasting blood glucose concentration ≥ 100 mg/dL; and 5) Systolic blood pressure ≥ 130 mm Hg or diastolic blood pressure ≥ 80 mm Hg and/or using antihypertensive medications
Chronic Kidney Disease (CKD): CKD stage 2 (estimated Glomerular Filtration Rate [eGFR]: 60-89 mL/min/1.73m^2^ with albuminuria) or CKD stage 3 (eGFR 30-59 mL/min/1.73m^2^)

**Figure S5. Prevalence of different combinations of metabolic risk factors among Jackson Heart Study participants with Cardiovascular-Kidney-Metabolic (CKM) syndrome stage 3**


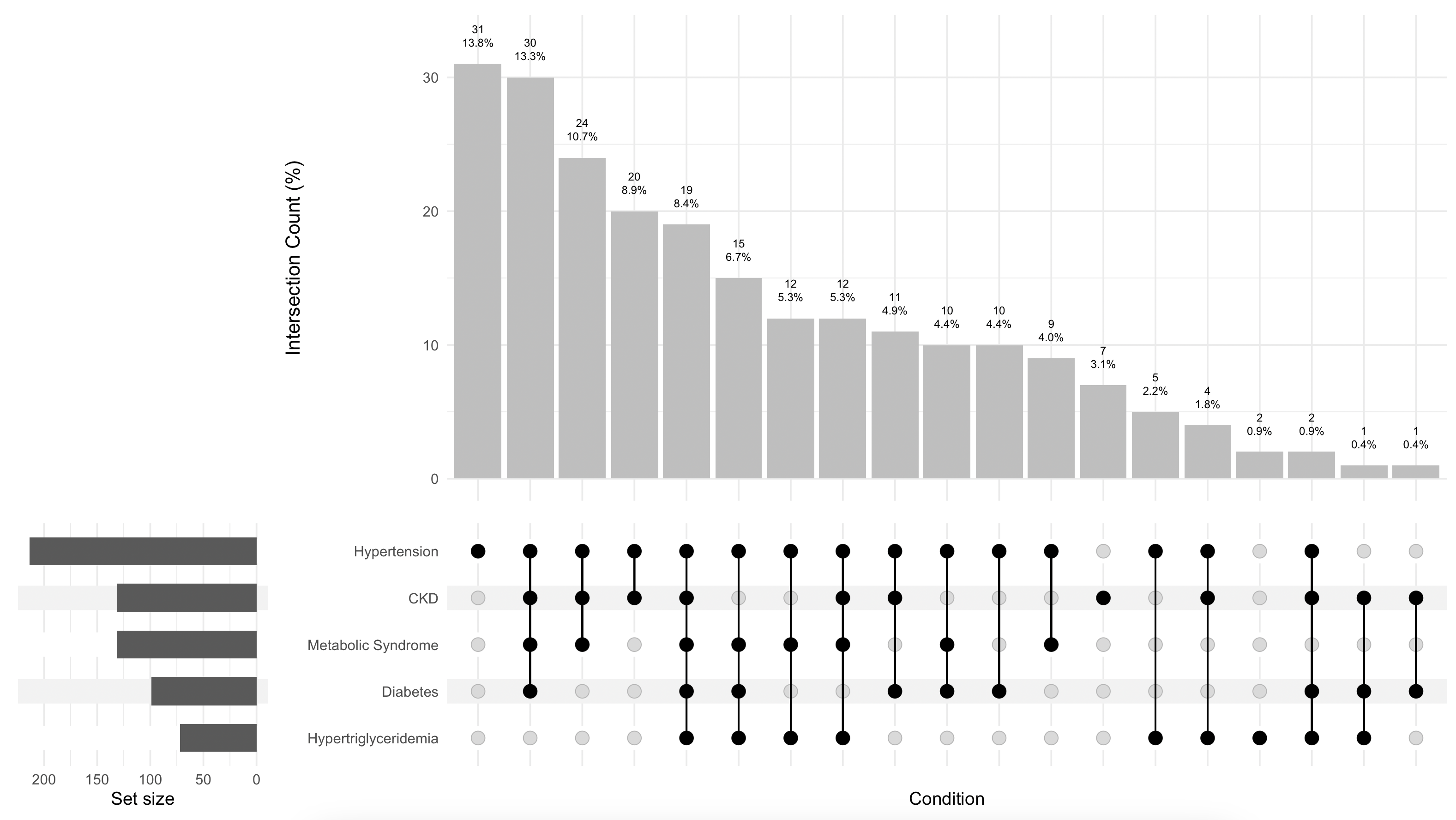


Hypertension: taking antihypertensive medication or having a systolic blood pressure ≥ 130 mm Hg or having a diastolic blood pressure ≥ 80 mm Hg
Diabetes: HbA1c ≥ 6.5% or a fasting blood glucose ≥ 126 mg/dL or using glucose-lowering medication.
Hypertriglyceridemia: serum triglycerides ≥ 125 mg/dL
Metabolic syndrome: If participants had 3 or more of the following 5 criteria they were considered to have the metabolic syndrome. 1) High serum triglyceride concentration (≥ 150 mg/dL); 2) High density lipoprotein (HDL) cholesterol < 40 mg/dL for men and < 50 mg/dL for women; 3) Waist circumference > 102 cm for men and > 88 cm for women; 4) Fasting blood glucose concentration ≥ 100 mg/dL; and 5) Systolic blood pressure ≥ 130 mm Hg or diastolic blood pressure ≥ 80 mm Hg and/or using antihypertensive medications
Chronic Kidney Disease (CKD): CKD stage 2 (estimated Glomerular Filtration Rate [eGFR]: 60-89 mL/min/1.73m^2^ with albuminuria) or CKD stage 3 (eGFR 30-59 mL/min/1.73m^2^)
